# Expression of Angiotensin II and Aldosterone in Radiation-induced Lung Injury

**DOI:** 10.7497/j.issn.2095-3941.2012.04.006

**Published:** 2012-12

**Authors:** Shuo Cao, Rong Wu

**Affiliations:** Department of Oncology, Shengjing Hospital of China Medical University, Shenyang 110022, China

**Keywords:** angiotensin II, aldosterone, transforming growth factor-β1, rats, lung, radiation injury

## Abstract

**Objective:**

Radiation-induced lung injury (RILI) is the most common, dose-limiting complication in thoracic malignancy radiotherapy. Considering its negative impact on patients and restrictions to efficacy, the mechanism of RILI was studied.

**Methods:**

Wistar rats were locally irradiated with a single dose of 0, 16, and 20 Gy to the right half of the lung to establish a lung injury model. Two and six months after irradiation, the right half of the rat lung tissue was removed, and the concentrations of TGF-β1, angiotensin II, and aldosterone were determined via enzyme-linked immunosorbent assay.

**Results:**

Statistical differences were observed in the expression levels of angiotensin II and aldosterone between the non-irradiation and irradiation groups. Moreover, the expression level of the angiotensin II-aldosterone system increased with increasing doses, and the difference was still observed as time progressed.

**Conclusions:**

Angiotensin II-aldosterone system has an important pathophysiological function in the progression of RILI.

## Introduction

Radiotherapy is used in the treatment of lung cancer, and 30%-40% of cancer patients benefit from it^[^^1^^]^. However, 20% of these patients develop radiation-induced pulmonary injury (RILI). The effectiveness of radiotherapy for thoracic malignancies is limited by the occurrence of RILI^[^^2^^]^, which has no effective treatment or prophylaxis yet^[^^3^^]^. RILI is usually divided into two stages, namely, early radiation pneumonitis and late fibrosis. From the animal data, an early overproduction of both pro-inflammatory and pro-fibrogenic cytokines exists during thoracic irradiation and sustained production in the development of acute and late pulmonary toxicities^[^^4^^-^^7^^]^. Studies have shown that cytokines related to the injury after irradiation include transforming growth factor-β (TGF-β), tumor necrosis factor (TNF-α), interleukin-1 (IL-1), interleukin-6 (IL-6), platelet-derived growth factor (PDGF), etc., among which the function of TGF-β1 is extensive. Angiotensin II and aldosterone are integral components of the renin-angiotensin-aldosterone system (RAAS) and are widespread in the heart, blood vessel wall, brain, lung, and other tissues and organs. Their potential pro-inflammatory properties have an important function in (causing or curing) organ fibrosis^[^^8^^-^^10^^]^ and radiation heart disease^[^^11^^]^, specifically with the ACE inhibitor that mitigates pulmonary injury caused by radiation^[^^12^^]^. With the hope of providing new research directions for the prevention and treatment of RILI, we hypothesized that RILI may increase locally produced angiotensin, aldosterone, and TGF-β1.

## Materials and Methods

### Animals

The experiments were performed using female albino Wistar rats (200-250 g). The animals were obtained from the Center for Experimental Animals at China Medical University (Shenyang, China) with a National Animal Use license number of SCXK-LN 2003-0009. All experiments were approved by the Animal Care and Use Committee at China Medical University, which complies with the National Institutes of Health Guide for the Care and Use of Laboratory Animals. All efforts were made to minimize the number of animals used and their suffering. Five animals were housed per cage at an environmental temperature of (24±1)°C and a 12/12 h light/dark cycles. The animals were fed with food and water *ad libitum*. The animals were randomly assigned into three groups (*n*=19 each), namely, the control group, the 16 Gy-irradiation treatment group, and the 20 Gy-irradiation treatment group. The control group received no irradiation, the irradiation treatment groups were irradiated at the right hemi-thoracic area by using a 10 MeV electron linear accelerator at a dose of 16 Gy and 20 Gy, respectively.

### Irradiation

The animals were anesthetized with an intraperitoneal injection of chloral hydrate at a dose of 0.3 mL/100 g prior to irradiation. Hemithoracic irradiation was performed on the right lung at a single dose of 16 Gy or 20 Gy. These doses have been shown to result in lung injury^[^^13^^-^^15^^]^. The left thorax, as well as the rest of the body, was shielded with 3 mm lead. Only 50% of the volume, which did not entail significant cardiac irradiation, was used in this study to exclude the indirect effects on pulmonary function. The animals were anesthetized and killed by cervical dislocation two and six months after irradiation. These periods have been proven to be sufficient for the development of RILI in rats.

### Tissue isolation

The right lungs were immediately removed after death without being perfused. The upper lobe of the right lungs was placed in fixative for histologic analysis and immunohistochemistry, whereas the right middle and lower lobes of the right lungs were quickly frozen for enzyme-linked immunosorbent assay (ELISA) analyses.

### Measurements

The levels of TGF-β1 were detected via ELISA by using a commercially available rat TGF-β1 ELISA Kit (RapidBio Lab, USA). Angiotensin II and aldosterone contents in the right middle and lower lobes were measured using commercial kits (RapidBio Lab, USA for angiotensin II; RapidBio Lab, USA for aldosterone). Standard curves were constructed, and the optical densities of samples were read from these curves (all values were within the calibration curve range).

### Histology

For histologic analysis, the upper lobe of the right lungs was fixed in 10% neutral-buffered formalin, and then embedded in paraffin. Tissue sections with a thickness of 4 µm were obtained, and were stained via Hematoxylin-Eosin (H&E) and Masson methods to determine pathological changes. The slides were examined via light microscopy. Fibrosis was defined as the thickened alveolar walls with superimposed collagen.

### Statistical analysis

Results are given as mean ± standard errors. The expression levels of TGF-β1 and aldosterone between groups were compared with the least significant difference test (LSD test). The expression level of angiotensin II was analyzed with the Duunett T3 test. Values of *P*<0.05 were considered significant. A SPSS13.0 statistical software was applied for statistical analysis.

## Results

**Figure 1:**
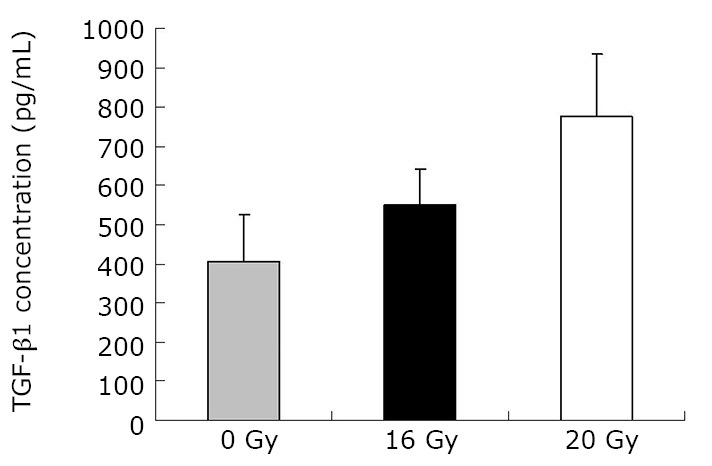
Concentration of TGF-β1 at two months.

**Figure 2:**
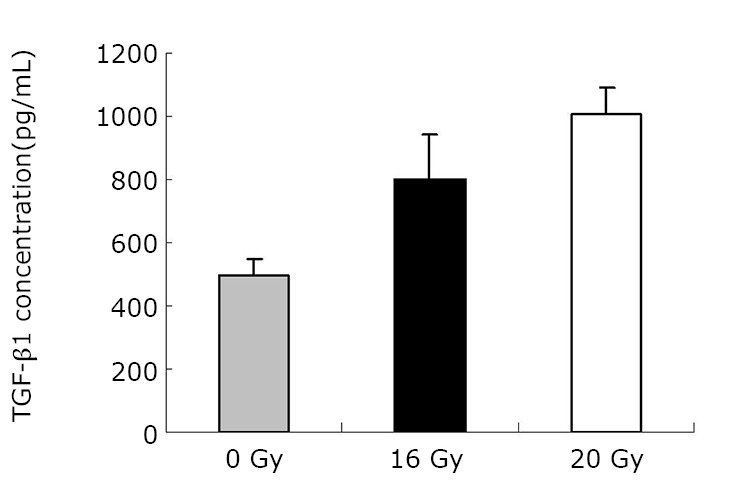
Concentration of TGF-β1 at six months.

**Figure 3:**
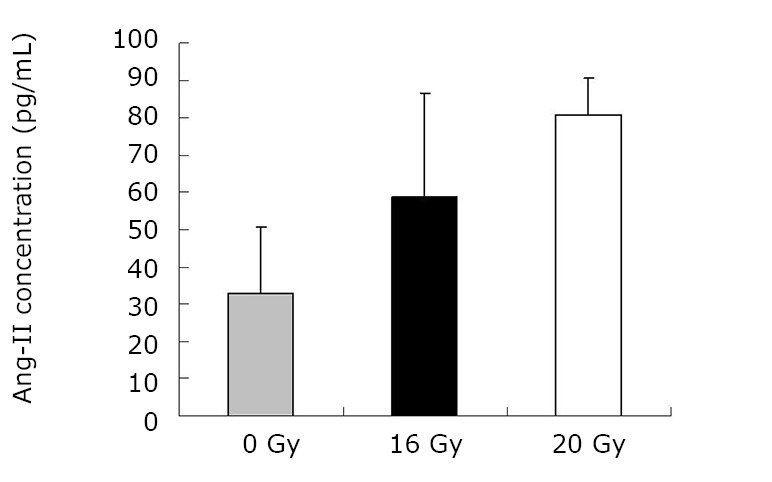
Concentration of angiotensin at two months.

**Figure 4:**
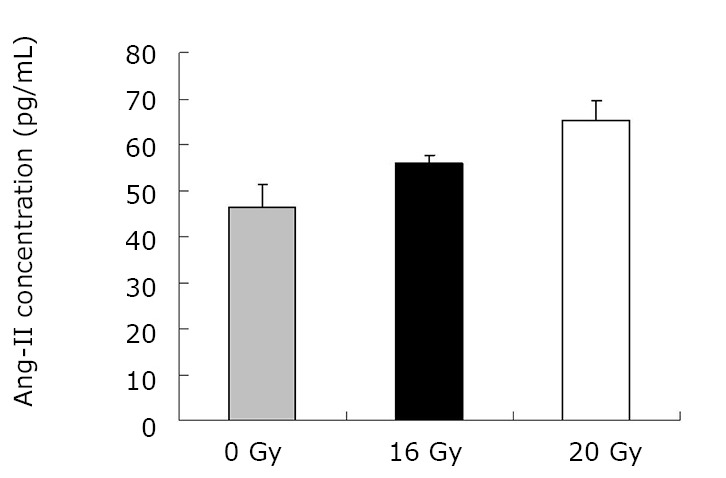
Concentration of angiotensin at six months.

**Figure 5:**
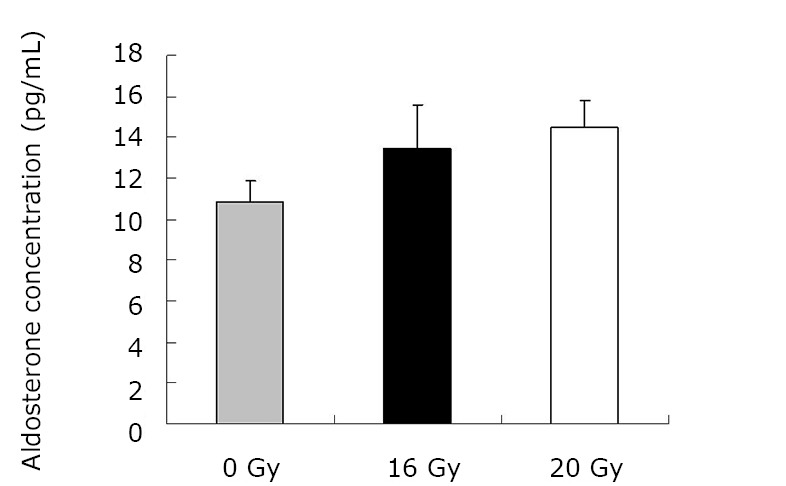
Concentration of aldosterone at two months.

**Figure 6:**
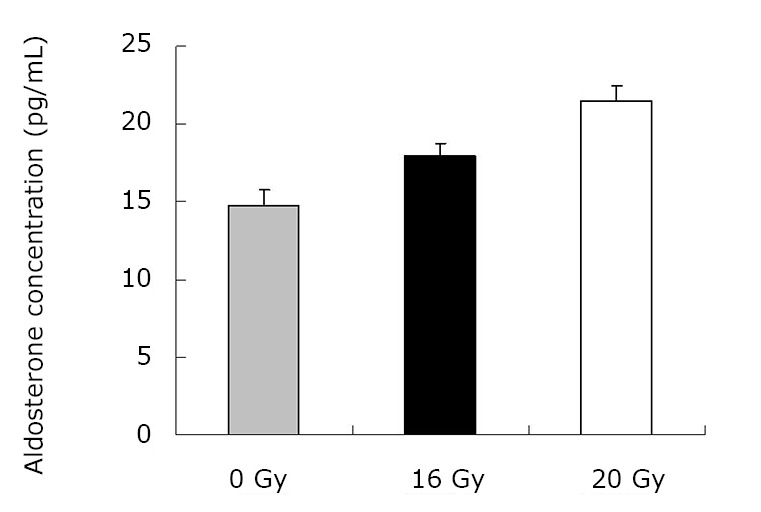
Concentration of aldosterone at six months.

**Figure 7:**
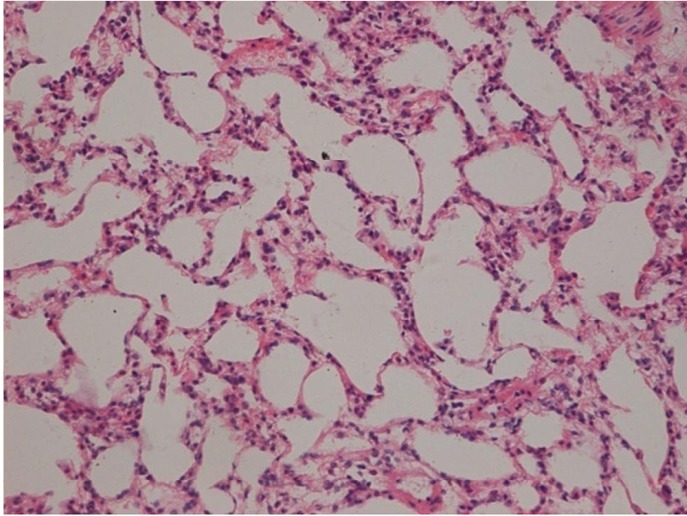
Histological tissue section and alveolar structural integrity area of the control group without alveolitis and pulmonary fibrosis (at two months), (H&E staining, ×200).

**Figure 8:**
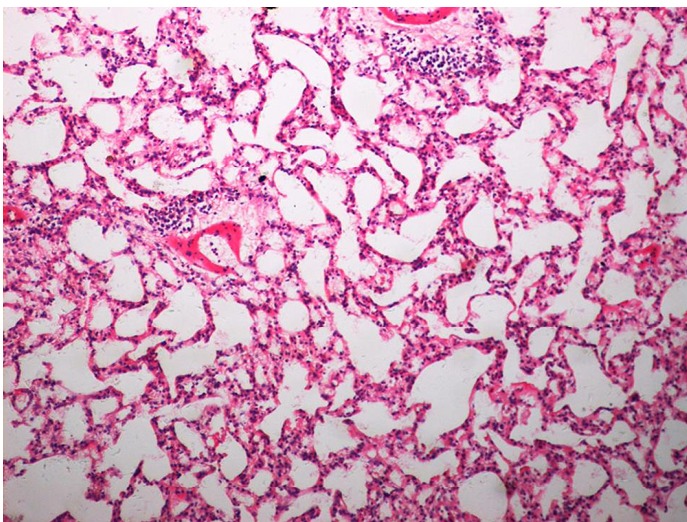
Histological tissue section and alveolar structural integrity area of the control group without alveolitis and pulmonary fibrosis (at six months), (H&E staining, ×200).

**Figure 9:**
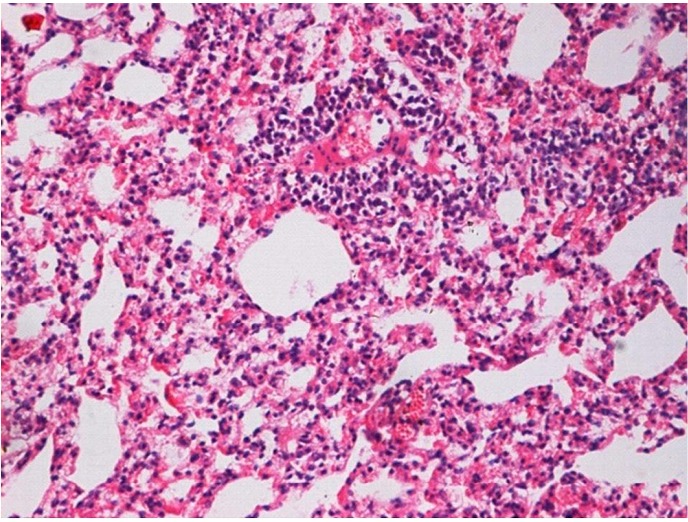
Histological tissue section of the irradiation group (16 Gy) with limited inflammation area (at two months), (H&E staining, ×200).

**Figure 10:**
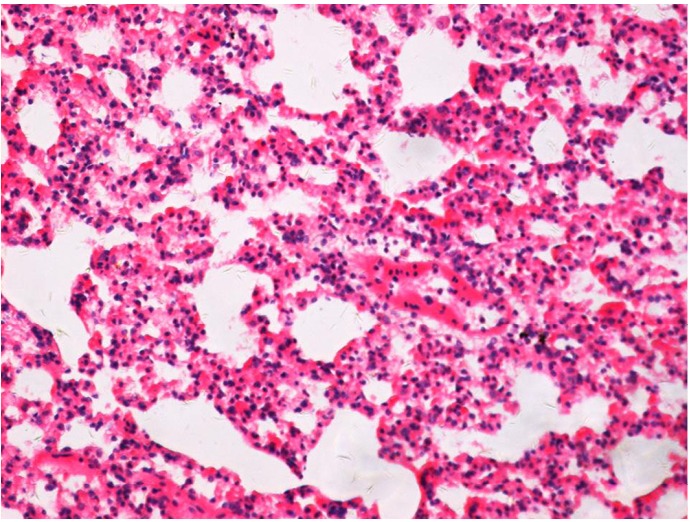
Histological tissue section of the irradiation group (16 Gy) with limited inflammation area (at six months), (H&E staining, ×200).

**Figure 11:**
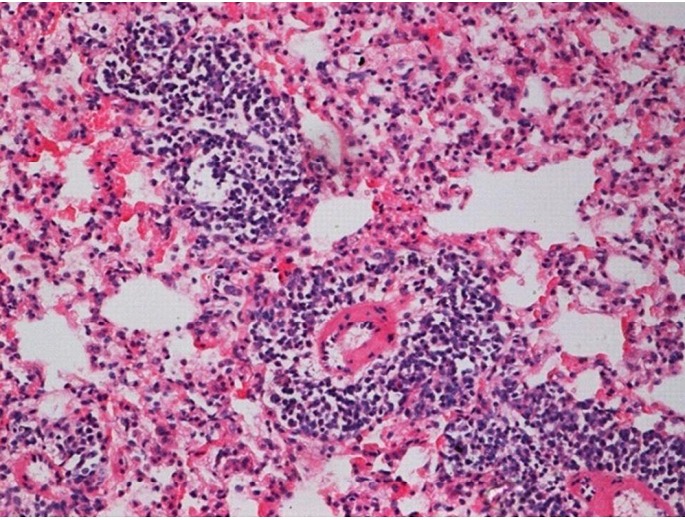
Histological tissue section of the irradiation group (20 Gy) with increased inflammation area (at two months), (H&E staining, ×200).

**Figure 12:**
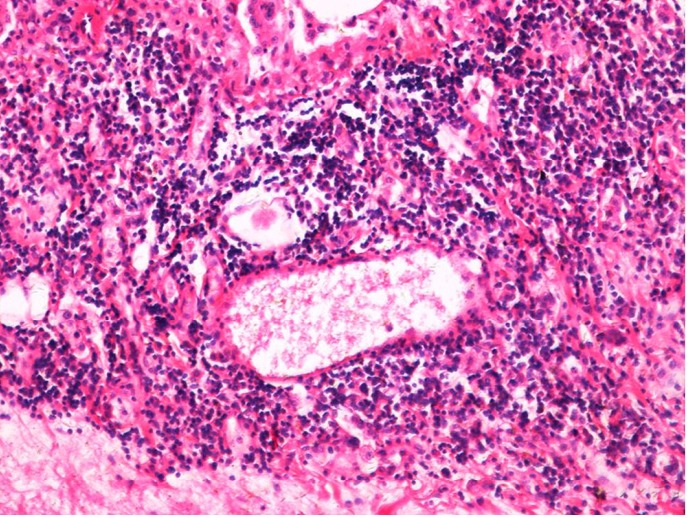
Histological tissue section of the irradiation group (20 Gy) with increased inflammation area (at six months), (H&E staining, ×200).

As demonstrated in **Table 1** and **Figure 1**, the mean TGF-β1 level (at two months) was (406.43±120.59) pg/mL for the control group, and (550.17±90.51) pg/mL and (774.20±160.77) pg/mL for the treated groups (16 Gy and 20 Gy, respectively). The rats that developed RILI showed a statistically significant higher level of TGF-β1 (*P*=0.005, *P*<0.001). The TGF-β1 level increased with increasing radiation dose, and the value for the 20 Gy group was higher than that for the 16 Gy group (*P*<0.001). However, **Table 2** and **Figure 2** show the TGF-β1 level at six months, where the mean level was (498.55±49.55)pg/mL, (802.23±136.71) pg/mL, (1004.59±86.89) pg/mL for the control and treated groups (16 Gy and 20 Gy), respectively. The concentrations for the treated groups were higher (*P*=0.003, *P*<0.001), and differences were detected between the two irradiation groups (*P*=0.037). The mean values of the expression of angiotensin II for two months are shown in **Table 3** and **Figure 3**. Significant differences in the expression level of angiotensin II were observed between non-irradiation and irradiation groups (*P*=0.005, *P*<0.001). Moreover, statistical differences were observed between the two irradiation groups (*P*<0.001). As shown in **Table 4** and **Figure 4**, the angiotensin II concentration in the irradiation groups was higher than that in the control group (*P*=0.006, *P*<0.001), and differences were also detected between the two irradiation groups (*P*=0.004). The mean values of the aldosterone expression at two months are shown in **Table 5** and **Figure 5**. The aldosterone concentration in the irradiation groups was higher than that in the control group (*P*=0.01, *P*<0.001), but significant differences were not detected between the two irradiation groups (*P*=0.131). **Table 6** and **Figure 6** show the aldosterone level at six months. The concentration in the treated groups was higher than that in the control group (*P*=0.006, *P*<0.001), and differences were detected between the two irradiation groups (*P*=0.03). Lung tissue inflammatory lesions and fibrosis were observed by H&E stains in the irradiation groups (**Figures 7****,****8****,****9****,****10****,****11****,****12**).

**Table 1:** Mean values of TGF-β1 (two months) in each group.

Group, pg/mL		Group, pg/mL	*P*
Group 1 (406.43±120.59)	*vs.*	Group 2 (550.17±90.51)	0.005
Group 1 (406.43±120.59)	*vs.*	Group 3 (774.20±160.77)	<0.001
Group 2 (550.17±90.51)	*vs.*	Group 3 (774.20±160.77)	<0.001

Group 1: Control Group (*n*=9); Group 2: Irradiation Group (16 Gy) (*n*=9); Group 3: Irradiation Group (20 Gy) (*n*=9).

**Table 2:** Mean Values of TGF-β1 (six months) in each group.

Group, pg/mL		Group, pg/mL	*P*
Group 1 (498.55±49.55)	*vs.*	Group 2 (802.23±136.71)	0.003
Group 1 (498.55±49.55)	*vs.*	Group 3 (1004.59±86.89)	<0.001
Group 2 (802.23±136.71)	*vs.*	Group 3 (1004.59±86.89)	0.037

Group 1: Control Group (*n*=10); Group 2: Irradiation Group (16 Gy) (*n*=10); Group 3: Irradiation Group (20 Gy) (*n*=10).

**Table 3:** Mean values of angiotensin-II (two months) in each group.

Group, pg/mL		Group, pg/mL	*P*
Group 1 (33.06±18.51)	*vs.*	Group 2 (58.79±27.91)	0.005
Group 1 (33.06±18.51)	*vs.*	Group 3 (81.23±9.81)	<0.001
Group 2 (58.79±27.91)	*vs.*	Group 3 (81.23±9.81)	<0.001

Group 1: Control Group (*n*=9); Group 2: Irradiation Group (16 Gy) (*n*=9); Group 3: Irradiation Group (20 Gy) (*n*=9).

**Table 4:** Mean values of angiotensin-II (six months) in each group.

Group, pg/mL		Group, pg/mL	*P*
Group 1 (46.50±4.89)	*vs.*	Group 2 (55.971±1.66)	0.006
Group 1 (46.50±4.89)	*vs.*	Group 3 (65.228±4.34)	<0.001
Group 2 (55.971±1.66)	*vs.*	Group 3 (65.228±4.34)	0.004

Group 1: Control Group (*n*=10); Group 2: Irradiation Group (16 Gy) (*n*=10); Group 3: Irradiation Group (20 Gy) (*n*=10).

**Table 5:** Mean values of aldosterone (two months) in each group.

Group, pg/mL		Group, pg/mL	*P*
Group 1 (10.82±1.04)	*vs.*	Group 2 (13.45±2.19)	0.01
Group 1 (10.82±1.04)	*vs.*	Group 3 (14.47±1.38)	<0.001
Group 2 (13.45±2.19)	*vs.*	Group 3 (14.47±1.38)	0.13

Group 1: Control Group (*n*=9); Group 2: Irradiation Group (16 Gy) (*n*=9); Group 3: Irradiation Group (20 Gy) (*n*=9).

**Table 6:** Mean Values of aldosterone (at six months) in each group.

Group, pg/mL		Group, pg/mL	*P*
Group 1 (14.77±1.03)	*vs.*	Group 2 (17.94±0.78)	0.006
Group 1 (10.82±1.04)	*vs.*	Group 3 (21.48±0.94)	<0.001
Group 2 (17.94±0.78)	*vs.*	Group 3 (21.48±0.94)	0.03

Group 1: Control Group (*n*=10); Group 2: Irradiation Group (16 Gy) (*n*=10); Group 3: Irradiation Group (20 Gy) (*n*=10).

## Discussion

The awareness of the risk of RILI is critical for treating patients especially now that chemoradiation is being used for treating lung cancer. In past years, researchers have focused on physical and biological parameters, and most of dosimetric factors showed an association with RILI^[^^15^^,^^16^^]^. Several patient- or treatment-specific factors have been identified as predictors for RILI. Consequently, the focus of recent research was directed towards gathering insights into the pathogenesis of RILI. These studies have revealed the possible link between plasma cytokine and the development of the disease. Thus, plasma cytokine can be used as a predictor for RILI^[^^17^^-^^19^^]^. However, the understanding of the pathogenesis of RILI remains unclear because of the lack of generally accepted methods in the prevention and treatment of RILI. RILI is a complex process that causes various cells in the lungs (endothelial and epithelial cells, as well as macrophages, pneumocytes, and fibroblasts) to produce a number of inflammatory and fibrogenic cytokines. Exposure to ionizing radiation triggers a cascade of genetic and molecular events, a series of cytokines and growth factor synthesis, and cell secretion through the cells, transmission of information between cells, and signal amplification to initiate visible and invisible clinical pathophysiological process. Several studies have shown that several cytokines have important functions in RILI, such as IL-1, IL-6, TNF-α, platelet-derived growth factor, TGF-β, surfactant apoproteins, and cell adhesion molecules (ICAM-1, E-selectin)^[^^20^^-^^24^^]^. Among these cytokines, TGF-β1 has the most functions in RILI. RILI is a continuous process of development, which begins from early inflammation to the late fibrosis phase. The proinflammatory phenotype is requisite to the appearance of fibrosis at these sites^[^^25^^,^^26^^]^. Fibrosis is the end result of chronic inflammatory reactions induced by a variety of stimuli without clear boundaries, such as persistent infections, chemical reactions, radiation, autoimmune reactions, allergic responses, and tissue injury. Current treatments for fibrotic diseases typically target the inflammatory response. In this research, the characteristic histological changes in the pneumonitis and fibrosis phases of the radiation response were observed.

Profibrogenic cytokine TGF-β1 is the most important among various biological markers in RILI because it contributes to increased tissue injury after exposure to an ionizing radiation. TGF-β1 has been implicated as a potent stimulator of fibrosis, and could promote the differentiation and proliferation of myofibroblasts and stimulate collagen synthesis^[^^25^^,^^27^^,^^28^^]^. TGF-β signaling may be a component of the early events leading to fibrosis, as well as a required factor in the fibrotic process^[^^29^^]^. Similar reports have indicated that in the early events after radiation, the TGF-β1 level in lung and serum increased. Treatment with TGF-β antagonists at the time of irradiation surprisingly reduces acute pneumonitis as well as the late phase fibrosis at six months after irradiation^[^^30^^-^^32^^]^. In this study, the TGF-β1 levels in the irradiated rat lung tissues markedly increased compared with those in the control group. Moreover, these levels increased over time. The results indicate that TGF-β1 may also be implicated in the progression of RILI. Similar results have been reported for the TGF-β1 expression in relation to the development of RILI^[^^33^^]^.

RAAS has been known to have important influences over vascular functions. However, angiotensin II and aldosterone are also involved in organ damage, i.e., pathologic tissue remodeling, which includes cellular hypertrophy, proliferation and/or migration, and extracellular matrix proliferation. Intensive studies have been carried out on angiotensin II as a pro-inflammatory mediator that stimulates the production of other growth factors and vasoconstrictors, transactivates several growth factor receptors, and influences cell contraction, cell growth, apoptosis, differentiation, and gene expression^[^^34^^-^^37^^]^. In both early and late phase results, the local angiotensin II levels increased in the treatment groups wherein the rats receiving an irradiation of 20 Gy had significantly higher angiotensin II levels. However, in the groups treated for six months, the angiotensin II levels decreased compared with those treated for two months. This result indicates that the angiotensin II level obviously increased in the early phase. Similar reports by Wang et al.^[^^38^^]^ revealed that angiotensin II, as a potential proinflammatory mediator, contributes to the development of LPS-induced acute lung injury. Additionally, several research have shown that the ACE inhibitor is an effective mitigator of pulmonary injury caused by survivable doses of radiation^[^^12^^]^. In addition, evidence that the local angiotensin II seems to have a more important function than the circulating angiotensin II in the regulation of angiotensin II-induced tissue injury is increasing^[^^39^^,^^40^^]^. The effect of angiotensin II is observed when it binds to high-affinity receptors on the cell surface. The AT1 receptor has been determined to have a more important function compared with angiotensin II, especially in angiotensin II-mediated cardiovascular diseases and lung inflammation^[^^38^^,^^41^^,^^42^^]^. Otsuka et al.^[^^43^^]^ reported that the pulmonary AT1 receptor expression obviously increased in a lung fibrosis model. The results by Rosenkranz S et al. demonstrated that angiotensin II stimulated TGF-β1 secretion and activation and enhanced TGF- β1 signaling *in vivo* and *in vitro*^[^^44^^-^^46^^]^. Thus, one path of injury may proceed through the generation of angiotensin II and results in the production of TGF-β1.

An increasing number of studies have clarified that aldosterone can directly injure various organs, such as the heart, blood vessel, and kidney^[^^47^^,^^48^^]^. Multiple studies have led to the hypothesis that aldosterone has a direct effect on the synthesis of extracellular matrix proteins, which may lead to the development of tissue fibrosis^[^^49^^]^. In several research, aldosterone, which has been determined to be in pathological states, exerts profibrotic effects by increasing the expression of TGF-β1^[^^50^^]^. Treatment with aldosterone/salt can induce a proinflammatory/fibrogenic phenotype, which is a coupling of an inflammatory response and the release of several proinflammatory mediators, which include an adhesion molecule, a chemokine, and a proinflammatory cytokine (ICAM-1, MCP-1, and TNF-β1, respectively). The aldosterone/salt-induced proinflammatory phenotype is a necessary requisite to the accumulation of fibrous tissue at vascular and nonvascular sites of injury in the heart^[^^25^^]^. Haruhiko et al.^[^^51^^]^ demonstrated that vascular cells are steroidogenic with their own responding system by detecting the CYP11B2 mRNA that encodes the key enzyme for the biosynthesis of aldosterone in both endothelial and smooth muscle cells cultivated from a human pulmonary artery. Locally produced aldosterone is likely to exert its effects on or in SMC in a paracrine, autocrine, or intracrine manner. Moreover, all components of the rennin-angiotensin system were expressed in the vascular wall^[^^36^^]^. In addition, Zhao et al.^[^^25^^]^ indicated that the aldosterone was involved in the angiotensin II-induced cardiac injury. However, similar to our research, other studies have also failed to find a relationship between aldosterone and RILI. In all irradiated groups of this study, a significant increase was found in the irradiated rats compared with those of the control group. However, no statistical differences were observed between the irradiated groups for two months. For rats treated for six months, the aldosterone levels increased, and the difference between the two irradiated groups were significant. This result indicates that aldosterone levels increased over time, and the difference between the irradiated groups is significant.

Although various cytokines are identified to have important functions in the pathogenesis of RILI, our results show that the changes in the angiotensin II-aldosterone system could be important factors in the development of radiation pneumonitis as well as effective predictors of the fibrosis. However, the pathological and physiological mechanisms of the relationship between the angiotensin II-aldosterone system and RILI need further investigation. Moreocer, therapies that target RAAS or TGF-β1 pathways might provide effective strategies to treat the inflammation or slow the progression of fibrosis in RILI.
